# Magnitude and dynamics of the T-cell response to SARS-CoV-2 infection at both individual and population levels

**DOI:** 10.3389/fimmu.2024.1488860

**Published:** 2025-01-07

**Authors:** Thomas M. Snyder, Rachel M. Gittelman, Mark Klinger, Damon H. May, Edward J. Osborne, Ruth Taniguchi, H. Jabran Zahid, Ian M. Kaplan, Jennifer N. Dines, Matthew T. Noakes, Ravi Pandya, Xiaoyu Chen, Summer Elasady, Emily Svejnoha, Peter Ebert, Mitchell W. Pesesky, Patricia De Almeida, Hope O’Donnell, Quinn DeGottardi, Gladys Keitany, Jennifer Lu, Allen Vong, Rebecca Elyanow, Paul Fields, Hussein Al-Asadi, Julia Greissl, Lance Baldo, Simona Semprini, Claudio Cerchione, Fabio Nicolini, Massimiliano Mazza, Ottavia M. Delmonte, Kerry Dobbs, Rocio Laguna-Goya, Gonzalo Carreño-Tarragona, Santiago Barrio, Luisa Imberti, Alessandra Sottini, Eugenia Quiros-Roldan, Camillo Rossi, Andrea Biondi, Laura Rachele Bettini, Mariella D’Angio, Paolo Bonfanti, Miranda F. Tompkins, Camille Alba, Clifton Dalgard, Vittorio Sambri, Giovanni Martinelli, Jason D. Goldman, James R. Heath, Helen C. Su, Luigi D. Notarangelo, Estela Paz-Artal, Joaquin Martinez-Lopez, Bryan Howie, Jonathan M. Carlson, Harlan S. Robins

**Affiliations:** ^1^ Adaptive Biotechnologies, Seattle, WA, United States; ^2^ Microsoft Research, Redmond, WA, United States; ^3^ Unit of Microbiology - The Great Romagna Hub Laboratory, Pievesestina ITALY and DIMES, University of Bologna, Bologna, Italy; ^4^ IRCCS Istituto Romagnolo per lo Studio dei Tumori (IRST) “Dino Amadori”, Meldola, Italy; ^5^ Immunotherapy, Cell Therapy and Biobank (ITCB), IRCCS Istituto Romagnolo per lo Studio dei Tumori (IRST) “Dino Amadori”, Meldola, Italy; ^6^ Immune Deficiency Genetics Section, Laboratory of Clinical Immunology and Microbiology, National Institute of Allergy and Infectious Diseases, National Institutes of Health, Bethesda, MD, United States; ^7^ Department of Immunology, Hospital 12 de Octubre, CNIO, Complutense University, Madrid, Spain; ^8^ Hematology Department, Hospital 12 de Octubre, CNIO, Complutense University, Madrid, Spain; ^9^ Laboratorio CREA, Department of Infectious and Tropical Diseases, and Medical Officer, ASST Spedali Civili di Brescia and University of Brescia, Brescia, Italy; ^10^ Department of Pediatrics and Centro Tettamanti-European Reference Network PaedCan, EuroBloodNet, MetabERN-University of Milano-Bicocca-Fondazione MBBM-Ospedale San Gerardo, Monza, Italy; ^11^ Department of Infectious Diseases, University of Milano-Bicocca-Ospedale San Gerardo, Monza, Italy; ^12^ The American Genome Center, Uniformed Services University of the Health Sciences, Bethesda, MD, United States; ^13^ Department of Anatomy, Physiology and Genetics, Uniformed Services University of the Health Sciences, Bethesda, MD, United States; ^14^ Swedish Medical Center, Seattle, WA, United States; ^15^ Division of Allergy and Infectious Diseases, University of Washington, Seattle, WA, United States; ^16^ Institute for Systems Biology, Seattle, WA, United States

**Keywords:** SARS-CoV-2, COVID-19, T cell, TCR repertoire, immune response, cellular immunity

## Abstract

**Introduction:**

T cells are involved in the early identification and clearance of viral infections and also support the development of antibodies by B cells. This central role for T cells makes them a desirable target for assessing the immune response to SARS-CoV-2 infection.

**Methods:**

Here, we combined two high-throughput immune profiling methods to create a quantitative picture of the T-cell response to SARS-CoV-2. First, at the individual level, we deeply characterized 3 acutely infected and 58 recovered COVID-19 subjects by experimentally mapping their CD8 T-cell response through antigen stimulation to 545 Human Leukocyte Antigen (HLA) class I presented viral peptides. Then, at the population level, we performed T-cell repertoire sequencing on 1,815 samples (from 1,521 COVID-19 subjects) as well as 3,500 controls to identify shared “public” T-cell receptors (TCRs) associated with SARS-CoV-2 infection from both CD8 and CD4 T cells.

**Results:**

Collectively, our data reveal that CD8 T-cell responses are often driven by a few immunodominant, HLA-restricted epitopes. As expected, the T-cell response to SARS-CoV-2 peaks about one to two weeks after infection and is detectable for at least several months after recovery. As an application of these data, we trained a classifier to diagnose SARS-CoV-2 infection based solely on TCR sequencing from blood samples, and observed, at 99.8% specificity, high early sensitivity soon after diagnosis (Day 3–7 = 85.1% [95% CI = 79.9–89.7]; Day 8–14 = 94.8% [90.7–98.4]) as well as lasting sensitivity after recovery (Day 29+/convalescent = 95.4% [92.1–98.3]).

**Discussion:**

The approaches described in this work provide detailed insights into the adaptive immune response to SARS-CoV-2 infection, and they have potential applications in clinical diagnostics, vaccine development, and monitoring.

## 1 Introduction

The adaptive immune response to infection includes both a cellular and humoral component. The cellular immune response is mediated by T cells, which play a role in direct killing of virus-infected cells via cytotoxic (CD8) T cells as well as helping to direct the overall immune response through helper (CD4) T cells. The humoral immune response also includes CD4 T cells which assist B cells to differentiate into plasma cells and subsequently produce antibodies specific to a targeted antigen. As T cells are involved in the early identification and clearance of viral infections by both cellular and humoral immunity, they are a desirable target for assessing SARS-CoV-2 exposure (1–5).

Healthy adults have ~10^12^ circulating T cells expressing approximately 10^7^ unique TCRs (6). This diversity allows the full repertoire of T cells to potentially recognize a wide variety of peptide antigens displayed by HLA molecules on the surface of cells. When a naïve T cell is activated in response to recognition of a cognate antigen presented by a specialized antigen presenting cell, it undergoes clonal expansion, resulting in an exponentially increasing number of genetically identical T cells. Due to the extreme sequence diversity possible among TCR rearrangements, particularly the TCR-beta chain, each observed TCR sequence is essentially a unique tag for a clonal lineage of T cells. Thus, the number of copies of each TCR sequence represents the number of T cells in that clonal lineage and provides information about the natural history of T-cell clonal expansions. Measuring the cellular immune response can provide a view into the state of the overall immune response, and several qualities of the adaptive cellular immune response suggest a T-cell-based assay may fulfill unmet clinical needs. In general, the T-cell immune response is: 1) Sensitive: T cells detect even a very small amount of antigen; 2) Specific: TCRs bind only to specific antigens; 3) Naturally amplified: T cells proliferate and clonally expand upon recognition of small quantities of specific antigen via their TCRs; 4) Systemic: T-cell clones circulate throughout the body in the blood; and 5) Persistent: a subset of T cells are maintained following clonal contraction in long term memory (7–11). The T-cell response is typically the first component of the adaptive immune response that can be measured, within days from initial pathogen exposure, and after clonal expansion and transition into memory can persist for years even when antibodies become undetectable. In the context of coronavirus infections, persistent T cells specific for SARS-CoV-1 have been routinely detected in studies in the years following the initial SARS outbreak (12, 13), including at least a decade after initial infection (14). Subjects show lasting memory T-cell populations to SARS-CoV-1 even as IgG antibodies and peripheral memory B cells become undetectable in a majority of convalescent subjects (13). Similarly, T cells responsive to the Middle East respiratory syndrome (MERS) coronavirus were observed in the absence of detectable antibodies (15).

Standard methods to assess the cellular immune response to a pathogen are based on T-cell recognition of target antigens. Conventional immune monitoring assays, including ELISpot and ICS, rely on functional T-cell responses and require live T cells, thus limiting standardization and throughput. The emergence of the COVID-19 pandemic has generated the urgent need for a scalable molecular assay to assess the T-cell response to SARS-CoV-2. In response, Adaptive Biotechnologies and Microsoft have applied previously developed platforms to create T-MAP™ COVID, a TCR sequence-based approach to quantitatively assess the T-cell response to SARS-CoV-2. This approach utilizes a multiplexed experimental platform to interrogate T-cell repertoires with large numbers of query antigens to identify SARS-CoV-2-specific TCRs in the context of HLA (16). We have deeply characterized 61 COVID-19 subject samples against 545 potential peptide antigens to profile the CD8 immune response. We have further sequenced 1,815 blood samples from 1,521 COVID-19 cases with Adaptive TCRβ immunosequencing in order to identify a robust set of SARS-CoV-2 specific CD4 and CD8 TCRs from a fixed number of blood cells (17, 18). All of these data are available as part of the public ImmuneCODE data release at https://clients.adaptivebiotech.com/pub/covid-2020 (19).

Taken together, these approaches allow the development of a map between TCR sequences and SARS-CoV-2 specific antigens, as well as the identification of public SARS-CoV-2 specific TCRs shared across individuals. This approach allows us to characterize many of the antigens involved in a T-cell immune response. We also capture a measure of the clonal breadth (the estimated proportion of distinct T-cell clonal lineages in a repertoire that are SARS-CoV-2 specific) and depth (related to the relative frequency of SARS-CoV-2-specific T-cell clones in a repertoire), as well as the dynamics of the cellular immune response to a SARS-CoV-2 infection over time. The exact antigens targeted are elucidated for several of these clones, which may allow for mapping a vaccine response in comparison to the response in a natural infection (8). Moreover, a collection of public SARS-CoV-2 TCRs form a robust diagnostic for recent or past infection of SARS-CoV-2, which is no longer highly relevant but was used by a number of health providers earlier in the pandemic.

## 2 Materials and methods

### 2.1 Clinical sample collection

Samples were collected based on each institution’s study protocol, as reviewed by their Institutional Review Board. All samples in this study were collected prior to September 2020. From all sources, whole blood samples were collected in K2EDTA tubes and were stored until being shipped to Adaptive as frozen whole blood, isolated PBMC or DNA extracted from either blood or PBMC for immune profiling analyses via the Adaptive immunosequencing assay and/or MIRA.

Samples provided by the NIAID were collected under approval by Comitato Etico Provinciale (protocol NP-4000), and by Comitato Etico, Ospedale San Gerardo Monza (protocol COVID-STORM). The Brescia study includes collection of discarded blood samples obtained from patients who were admitted at ASST Spedali Civili Brescia following positive nasopharyngeal swab for SARS-CoV-2 infection. Samples were obtained from all patients admitted to the hospital, as long as discarded material was available. Patients in Monza were enrolled when they were admitted to the San Gerardo Hospital in Monza, criteria for enrollment required a positive COVID-19 PCR test.

Samples provided by Hospital 12 de Octubre were collected under approval by Comite Etico del Hospital 12 de Octubre, Madrid IC (protocol 20/161). Participants were recruited at Hospital Universitario 12 de Octubre from inpatient and hospital workers with a positive COVID-19 PCR test.

Samples provided by Swedish-ISB were collected under approval by the Providence St. Joseph’s Health system IRB (STUDY2020000175). Study participants were recruited at clinics associated with Swedish Medical Center with a confirmed diagnosis by SARS-CoV-2 PCR or persons under investigation (with PCR pending) with >3 diagnostic criteria. SARS-CoV-2 PCR was performed at enrollment to confirm diagnosis.

Samples provided by IRST and AUSL Romagna were collected under approval by CEROM (IRSTB113). Specifically, remnants of whole blood samples from diagnostic procedures of SARS-CoV-2 nasopharyngeal swab positive patients were stored frozen at −20°C before shipment to Adaptive.

Whole blood samples from DLS (Discovery Life Sciences, Huntsville, AL) were collected under Protocol DLS13 for collection of remnant clinical samples. All DLS subjects had tested positive for SARS-CoV-2 viral exposure by an Abbott RealTime SARS-CoV-2 RT-PCR assay.

From Bloodworks Northwest (Seattle, WA), volunteer donors recovered from COVID-19 were consented and collected under the Bloodworks Research Donor Collection Protocol BT001. Samples were processed for PBMC and donor data reported by the Biological Products division of Bloodworks NW under standard operating procedures. Inclusion criteria for samples collected by Bloodworks included age of at least 18 years old, weight of more than 110 lbs, a diagnosis of SARS-CoV-2 infection, at least 28 days since positive screening or days since last symptoms or a negative SARS-CoV-2 PCR test, and a provision of informed consent to participate in the study.

Controls were selected from primarily healthy controls drawn before 2020 by Diagnostic Laboratory Services, as well as other non-COVID studies. These samples are presumed negative and include collections during seasons with high prevalence of vaccination against, and/or infection with, the influenza A/B viruses and seasonal coronavirus(es) in order to exclude potential cross-reactivity.

### 2.2 ImmuneRACE sample collection and serology testing

The ImmuneRACE study (20) is a prospective, single group, multi-cohort, exploratory study of participants exposed to, infected with, or recovering from COVID-19 (NCT04494893). Participants from across the United States were consented and enrolled via a virtual study design, with cohorting based on participant-reported clinical history following the completion of both a screening survey and study questionnaire. All participants provided informed consent for sample collection and metadata use. Whole blood, serum, and a nasopharyngeal or oropharyngeal swab were collected from participants by trained mobile phlebotomists. The study was approved by Western Institutional Review Board (WIRB reference number 1-1281891-1, Protocol ADAP-006).

In this research, samples were selected from the first 100 individuals with self-reported COVID-19 based on an RT-PCR SARS-CoV-2 test from the acute and recovered cohorts as well as 23 individuals from the exposed cohort who at the time of enrollment and study questionnaire were within 2 weeks of exposure to someone diagnosed with COVID-19, asymptomatic, and not diagnosed with COVID-19. All of these samples were collected prior to September 2020. Whole blood samples were processed identically to other studies for the Adaptive immunosequencing assay. Additionally, serum samples were tested by Covance/LabCorp using two different EUA approved assays: 1) Elecsys^®^ Anti-SARS-CoV-2; Roche: qualitative detection of high affinity antibodies to SARS-CoV-2 including all isotypes, but preferentially detects IgG antibodies (https://www.labcorp.com/tests/164068/sars-cov-2-antibodies); and 2) SARS-CoV-2 Antibody, IgG; LabCorp: qualitative detection of IgG antibodies to SARS-CoV-2 (https://www.labcorp.com/tests/164055/sars-cov-2-antibody-igg).

### 2.3 Viral peptide selection

Using the NCBI genome reference for SARS-CoV-2 (RefSeq accession: NC_045512.2), a list of candidate 9–10AA long peptides from across the whole viral genome was identified based on predicted affinity (<1% rank) using NetMHCpan version 4.1 (21, 22) to common HLA-A and -B alleles as determined in the Allele Frequency Net Database (23). An additional 121 peptides were added to this list from (24), which identified candidate epitopes conserved between SARS-CoV-1 and SARS-CoV-2 and optimized for global HLA coverage. The final set of peptides included candidate epitopes for most common HLA alleles across the globe: A*01:01, A*02:01, A*02:07, A*03:01, A*11:01, A*23:01, A*24:02, A*31:01, A*33:01, A*33:03, A*68:01, B*07:02, B*08:01, B*13:01, B*15:01, B*15:02, B*18:01, B*27:05, B*35:01, B*40:01, B*44:02, B*46:01, B*51:01, B*58:01, C*14:02, C*15:02. Peptides were synthesized by GenScript (Piscataway, NJ). The complete list of peptides is in **Supplementary Table 1**.

The 545 peptides were then pooled in a combinatorial fashion as described previously (16); peptides that were overlapping or in close proximity in the viral proteome were grouped together into antigen sets. Each antigen set was then placed in a subset of 6 unique pools out of 11 pools; hereto after referred to as its occupancy. In order to estimate an empirical false discovery rate and gauge assay quality, we purposefully left > 40% of the unique occupancies empty to assess the rate at which clones are spuriously sorted and detected in 6 pools with no query antigen present.

Phylogenetic context of candidate epitopes was assessed using a customized BLAST database of 55 RefSeq coronavirus genomes across the Coronaviridae family (25). BLAST searches were optimized for short sequence queries using the “-task blastp-short” argument and all full-length, exact matching TCRs were used to assess the phylogenetic placement of each candidate epitope. Using the taxonomic annotations available from the NCBI taxonomy browser, the most recent common ancestor was defined as the most recent taxonomic node shared by all terminal taxa that shared an exact match to the epitope. Each epitope was also assessed for its homology to each of 4 endemic human coronaviruses: Human coronavirus 229E, Human coronavirus HKU1, Human coronavirus NL63, and Human coronavirus OC43 in order to explore the role of cross-reactivity in T cell responses.

### 2.4 Antigen stimulation experiments (MIRA)

Antigen-specific TCRs were identified using the Multiplex Identification of T-cell Receptor Antigen Specificity (MIRA; 16). For these MIRA from 61 COVID-19 subjects, bulk T cells from 5 to 40 million PBMCs were first polyclonally expanded with anti-CD3 (Biolegend clone OKT3, San Diego, CA) at 30 ng/ml, IL-2 (Biolegend, San Diego, CA) at 20 ng/ml, and IL-15 (Biolegend, San Diego, CA) at 5 ng/ml for 8–13 days. Equal aliquots of T cells post-expansion were then incubated with the 11 peptide pools outlined above at 0.5 ug/ml (per peptide) at 37°C. After expansion, there were typically 1-2 billion T cells from each subject. At 18 hours, cells were harvested and stained with antibodies for magnetic enrichment based on CD137 (Miltenyi Biotec, Gaithersburg, MD) and subsequent sorting by flow cytometry. Sorted T cells were then washed and suspended in PBS containing FBS (2%), 1mM EDTA and 4,6-diamidino- 2-phenylindole (DAPI) for exclusion of non-viable cells. Cells were acquired and sorted using a FACS Aria (BD Biosciences) instrument. The mean number of sorted cells from the peptide pool incubations (M=161,548; SD=346,745) was higher than that of the no-peptide control (M=7,226; SD=19,008). Sorted antigen-specific (CD3+CD8+CD137+) T cells were pelleted and lysed in RLT Plus buffer for nucleic acid isolation. RNA was then isolated using AllPrep DNA/RNA mini and/or micro kits, according to manufacturer’s instructions (Qiagen). RNA was reverse transcribed to cDNA using Vilo kits (Life Technologies), and TCRβ amplification performed using the Adaptive immunosequencing assay described below.

After immunosequencing, we examined the behavior of T-cell clonotypes by tracking read counts across each sorted pool. True antigen-specific clones should be specifically enriched in a unique occupancy pattern that corresponds to the presence of one of the query antigens in 6 pools. We have reported on methods to assign antigen specificity to TCR clonotypes previously (16). In addition to the previously published methods, we also developed a non-parametric Bayesian model to compute the posterior probability that a given clonotype is antigen specific. This model uses the available read counts of TCRs to estimate a mean-variance relationship within a given experiment as well as the probability that a clone will have zero read counts due to incomplete sampling of low frequency clones. Together, this model takes the observed read counts of a clonotype across all 11 pools and estimates the posterior probability of a clone responding to all possible 11 choose 6 addresses and an additional hypothesis that a clone is activated in all pools (truly activated, but not specific to any of our query antigens). To define antigen specific clones, we identified TCR clonotypes assigned to a query antigen from this model with a posterior probability ≥ 0.7. Further details of the Bayesian model are provided in the **Supplementary Methods**.

### 2.5 Immunosequencing of TCR repertoires

For blood or PBMC samples, genomic DNA was extracted from either peripheral blood mononuclear cells or from peripheral blood samples using the Qiagen DNeasy Blood Extraction Kit (Qiagen). As much as 18 μg of input DNA was then used to perform immunosequencing of the CDR3 regions of TCRβ chains using the Adaptive immunosequencing assay. Briefly, input DNA was amplified in a bias-controlled multiplex PCR, followed by high-throughput sequencing. Sequences were collapsed and filtered in order to identify and quantitate the absolute abundance of each unique TCRβ CDR3 region for further analysis as previously described (6, 17, 18). In order to quantify the proportion of T cells out of total nucleated cells input for sequencing, or T cell fraction, a panel of reference genes present in all nucleated cells was amplified simultaneously (26).

### 2.6 Characterization of the T-cell response with MIRA

In two separate analyses, each subject’s response to the antigens presented by the MIRA panel was summarized by the fraction of T cells responding to each protein, or to each antigen. Donors were clustered with average-linkage hierarchical clustering into five clusters (number of clusters chosen by visual inspection). For antigen-based clustering, only the 50 antigens present in the largest numbers of donors were used. 47 of the 61 donors, spread across the three large clusters, had HLA typing available. Association of each HLA with each antigen-based cluster was assessed with a one-sided Fisher’s Exact Test, using all available HLA typing.

### 2.7 Enhanced TCR sequence discovery and classification from case/control studies

Public TCRβ amino acid sequences (“enhanced sequences”) were associated with SARS-CoV-2 infection as described previously (27). Briefly, one-tailed Fisher’s exact tests were performed on all unique TCR sequences comparing the presence in SARS-CoV-2 positive samples with negative controls. Unique sequences were defined by their V gene, J gene, and CDR3 amino acid sequence. For subjects with longitudinal sampling, only the latest available sample was used.

Enhanced sequences were turned into a classifier predicting current or past infection with SARS-CoV-2 using a simple two feature logistic regression with dependent variables E and N, where E is the number of unique TCRβ DNA sequences that encode an enhanced sequence and N is the total number of unique TCRβ DNA sequences in that subject.

The significance threshold used to define the enhanced sequence set was chosen to maximize out-of-sample classification accuracy using 5-fold cross validation. In all cases described, the model identified p<0.001 as an optimal threshold, though the results were largely insensitive to the specific threshold chosen (data not shown).

In the final diagnostic classifier, an additional step was added to filter enhanced sequences that were common in the negative control samples as follows: first, a model was built with the initial set of enhanced sequences. Predictions were made on the training set to identify false positive control samples (model score >.35). Sequences that were present in two or more false positive control samples were removed from the enhanced sequence set before the final model was trained. The number of control samples a sequence was present in before exclusion and the score threshold for defining false positives were obtained by maximizing out-of-sample classification accuracy using 5-fold cross validation.

### 2.8 The breadth and depth of a disease-specific T-cell response

To summarize the extent to which a set of sequenced T cells is specific to a disease or set of antigens, we define the quantities clonal breadth *B* and clonal depth *D* as follows. For a given repertoire *j*, let *N_j_* be the number of unique TCR DNA sequences in the repertoire; $t_{ij},\;i=1,\ldots ,N_{j}$ , be the estimated number of T cells that have TCRβ DNA sequence *i* (assumed to derive from the same progenitor cell); and $M_{j}=\sum_{i}t_{ij}$ be the total number of T cells sequenced by the assay.

Then, for a given set of sequences 𝒟, the *clonal breadth of j with respect to* 𝒟 is defined to be


(1) $$B_{j}=N_{j}^{-1}\sum_{i\in D}I(t_{ij}>0)$$

where *I* is the indicator function and the summation is over all clones in 𝒟. That is, clonal breadth is the proportion of lineages in the repertoire that are mapped to the disease as defined by 𝒟.

Clonal depth is similar, but it attempts to capture the extent of clonal expansion of each lineage. Because the observed number of DNA templates derived from the same progenitor clone, *t_ij_*, is the result an exponential growth process, we use as our base measure of depth a number that is proportional to the estimated number of clonal generations that lineage *i* went through, $g_{ij}=log_{2}\left(1+t_{ij}\right)$ . Then the *clonal depth of j with respect to* 𝒟 is defined to be


(2) $$D_{j}=\sum_{i\in D}g_{ij}-log_{2}\left(M_{j}\right)$$

which estimates the relative number of clonal expansion generations across the TCRs in 𝒟, normalized by the total number of TCRs sequenced in the assay.

Error estimates on clonal breadth are derived starting from the assumption of Poisson error on the counting statistics comprising both the numerator $\sum_{i\in D}I(t_{ij}>0)$ and denominator *N_j_*. For clonal breadth, the full error on the quotient quantity $B=N_{j}^{-1}\sum_{i\in D}I(t_{ij}>0)$ is then given by


(3) $$\delta B_{j}=B\sqrt{\frac{1}{N_{j}}+\frac{1}{\sum_{i\in D}I(t_{ij}>0)}}$$

For clonal depth, errors are estimated starting from the same assumption of Poisson counting errors on both template counts for individual clones *t_i_* as $\delta t_{i}=\sqrt{t_{i}}$ and on total templates *M_j_* as $\delta M_{j}=\sqrt{M_{j}}$ . This error is then propagated to *g_ij_* as $\delta g_{ij}=\frac{\sqrt{t_{i}}}{\left(1+t_{i}\right)*log_{2}}$ . Adding in quadrature, the errors on the *g_ij_* along with the error on the normalization term $\delta log_{2}M_{j}=\frac{\sqrt{M_{j}}}{M_{j}log_{2}}$ gives the final uncertainty in the depth as


(4) $$\delta D_{j}=\sqrt{\sum_{i\in D}{\left(\delta g_{ij}\right)}^{2}+{\left(\delta log_{2}M_{j}\right)}^{2}}$$

## 3 Results

### 3.1 Identification of SARS-CoV-2-specific TCRs from COVID-19 subjects

To directly characterize the CD8 T-cell response to SARS-CoV-2, we applied MIRA (Multiplex Identification of T-cell Receptor Antigen Specificity), which maps TCRs to antigens at high scale and specificity (16). 545 query peptides derived from across the SARS-CoV-2 genome were selected from HLA-I NetMHCpan predictions across multiple representative HLA types (21, 22). These peptides were synthesized and assigned either individually or as groups of related peptides to one of 269 unique MIRA pools or “addresses” as described in the Materials and Methods.

**Figure 1:**
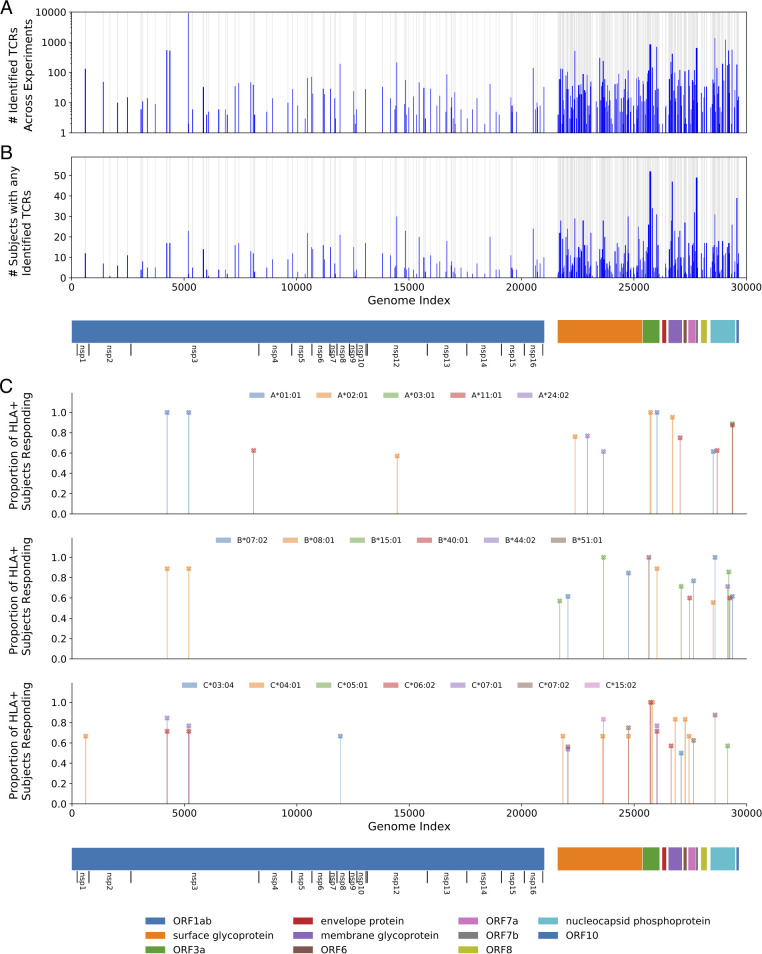
Magnitude and immunodominance of T-cell response to hundreds of potential SARS-CoV-2 antigens. **(A)** The count of identified TCRs across experiments at each antigen position in the viral genome. **(B)** A similar representation for the count of subjects that had at least one TCR identified in the data at that antigen position. The blue bars represent these counts while the gray background indicates the areas covered by the tested antigens. **(C)** The proportion of individuals with a given HLA that respond to a given antigen, restricting to immunodominant antigens. For this figure, we define *response* to mean a MIRA experiment using a subject who expresses the given HLA and for which the number of identified TCRs is more than two-fold higher than the median number observed for experiments with donors who do not express that HLA. Only HLAs that were observed in at least five donors are considered, and only HLA-antigen pairs with at least 50% response rates and significant median-fold enrichment are shown. Note that no correction was made for HLA linkage disequilibrium. Detailed data and significance tests are available in **Supplementary Table 4**. The 11 open reading frames from the virus are indicated below the plots, including extra notation for the 16 nonstructural proteins (nsp) encoded by ORF1ab.

MIRA was performed on T cells derived from PBMCs collected from 3 acutely infected and 58 convalescent COVID-19 subjects. Overall, 23,179 unique SARS-CoV-2 specific CD8 TCRs were identified 25,442 times across all experiments. The identified TCRs mapped to 260 of the 269 pools, representing antigens from across the viral proteome (**Figure 1**); the proteome was tiled densely outside of ORF1ab, as shown by the grey bars highlighting the positions of selected antigens. Strong immune responses (assessed by total number of TCRs, **Figure 1A**) as well as common immune responses (assessed by number of subjects with response to an antigen, **Figure 1B**) were observed across the viral proteome.

**Figure 2:**
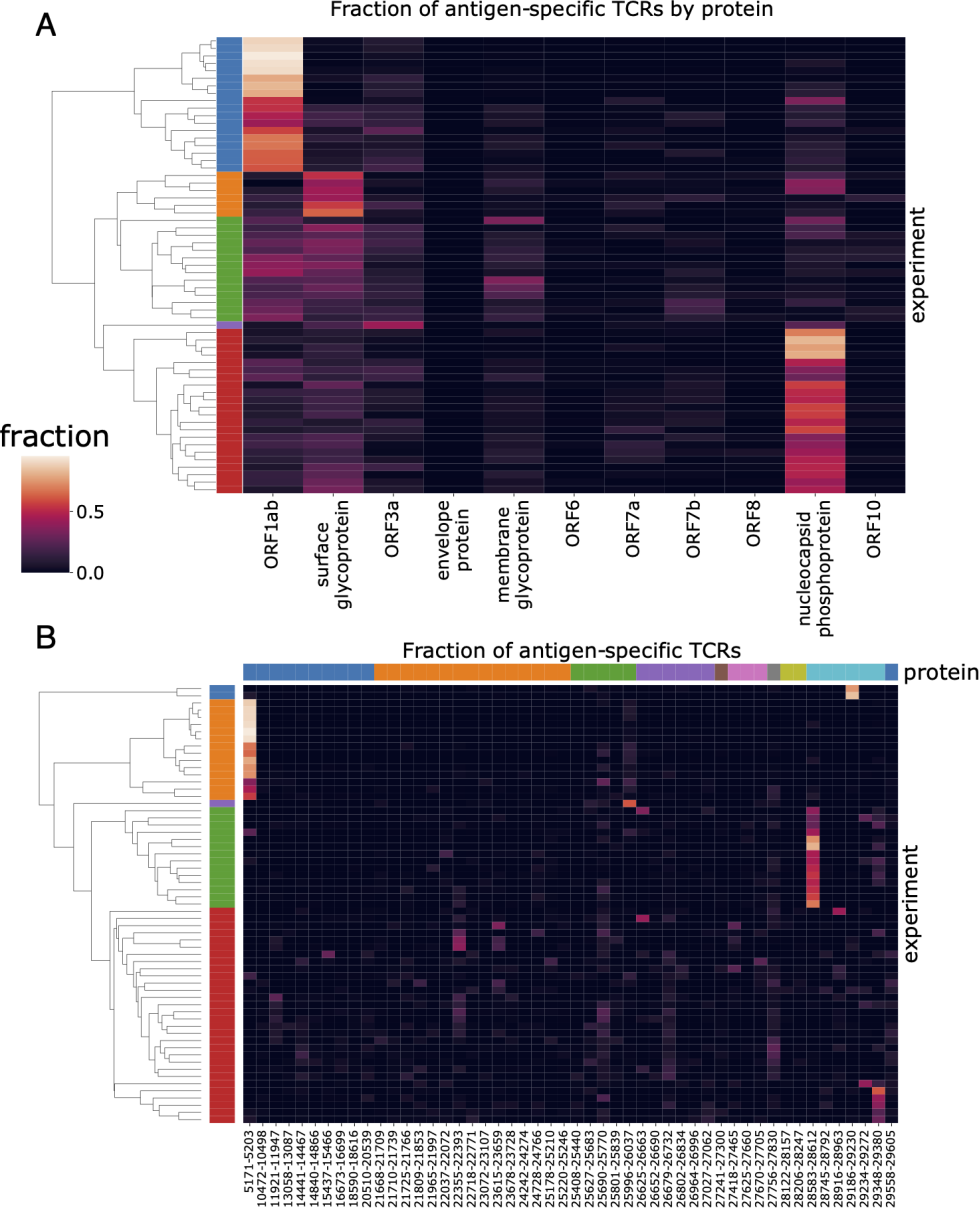
Patterns of antigen-TCR reactivity reveal immunodominance of some antigens. Clustergram plots of the **(A)** protein-level and **(B)** antigen-level signal across subjects. Each of the rows in the plot represents a distinct subject from the MIRA experiments; the left side label shows coloration for the top five subject clusters. In panel **(A)**, the columns represent the 11 viral proteins in viral genome order. In panel **(B)**, the columns represent the 50 antigens having the most donors with one or more TCRs reacting to them. They are sorted in viral genome order and colored by protein at the top. (Note that clustering is done independently for each panel to show the five farthest-separated clusters, and subject sets will vary by color across panels.).

We then explored the diversity of TCRs identified by MIRA across all the subjects by protein and by antigen. **Figure 2A** shows a clustergram of the protein-level response by subject, normalized to show the fraction of total TCRs identified per target. **Figure 2B** shows a similar analysis at the antigen-level, showing the 50 antigen locations with the most total TCRs observed across all subjects. A complete representation of the TCRs by antigen location is given in **Supplementary Table 1**. Preliminary analyses indicate these response data were heavily skewed by antigen, with 70% of all TCR mappings accounted for by 14 antigen pools (**Supplementary Table 1**). Similarly, responses to 8 antigens were observed in over half of the COVID-19 subjects’ MIRAs, suggesting these epitopes are frequently targeted during natural infection (**Supplementary Table 1**). While the TCRs binding these antigens are collectively common, they remain highly diverse: within each of these 8 antigens, no large clusters of TCRs with consistent CDR3 motifs were observed (**Supplementary Table 2**), and only a few TCRs were public enough to appear in multiple MIRA experiments (**Supplementary Table 1**) or in VDJdb (**Supplementary Table 3**; 28).

Our results suggest that in many subjects the immune response is dominated by a large number of distinct T cells against just a few epitopes, which may result from distinct HLA presentation. **Figure 2A** shows about 30% of subjects (first cluster, blue) have a predominantly ORF1ab-directed response in terms of total distinct T-cell clones, which is primarily explained by the single peptide HTTDPSFLGRY. Similarly, about 35% of subjects (fifth cluster, red) have a predominantly nucleocapsid phosphoprotein response, represented by at least two dominant antigen positions. Another cluster (third cluster, green) shows a more distributed response across multiple proteins/antigens while the second cluster in orange has stronger surface glycoprotein response.

An HLA association analysis to the identified antigen-level clusters (**Figure 2B**) was performed using Fisher’s exact test. The second cluster (orange) is primarily explained by TCRs associated with ORF1ab:5171–5203 (single peptide HTTDPSFLGRY). There are 12 subjects in this cluster with HLA typing available; all 12 have HLA-A*01:01 demonstrating significant enrichment (p=2e-10) for this allele considering only 13 subjects have this allele in this dataset. This peptide is predicted to be presented by HLA-A*01:01 by NetMHCpan. Similarly, the fourth cluster (green) contains 11 cases with HLA typing and all 11 subjects (out of 13 in this dataset) have HLA-B*07:02 (p=4e-9). There are two overlapping peptides in this address (LSPRWYFYY and SPRWYFYYL); the latter is predicted to be presented by HLA-B*07:02 by NetMHCpan.

Beyond this cluster-focused analysis, putative HLA restriction has been attributed to each of these pools using a Mann-Whitney’s U test over the number of mapped TCRs per experiment (**Supplementary Table 4**), identifying 41 strong associations (p < 0.01) between antigens and HLA alleles. Some of these associations were with HLA alleles that were not included in the epitope selection process, particularly HLA-C alleles; of the 25 HLA-C associations, 10 antigens were classified as weak or strong binders by NetMHC 4.1, and 10 of the remaining 15 were strongly associated with another HLA allele, likely due to linkage disequilibrium within the HLA locus (**Supplementary Table 4**). For 18 alleles, we identified at least one putative immunodominant epitope, which we defined as an HLA-antigen pair for which at least 50% of individuals with that allele respond to the antigen (see **Figure 1C** for details and definitions). These results are consistent with other recent reports of strong HLA-dependent CD8 T-cell responses to specific antigens (29, 30). These assignments and emerging immunodominance hierarchies will be further explored in later work as we continue to perform MIRA on cases and controls.

To assess the potential for T cell responses to be enhanced through cross-reactivity to common pathogens, we evaluated MIRA yields for epitopes that share direct homology to endemic human coronaviruses. In a comparison of homologous *vs*. non-homologous epitopes, we found positive but non-significant trends in the number of TCRs yielded (Kruskal-Wallis p=0.134) and the number of experiments with at least one TCR identified (Kruskal-Wallis p=0.112). These results are consistent with other published work demonstrating cross-reactivity of T cell responses between endemic coronaviruses and SARS-CoV-2 (31). Information about antigen homology between coronaviruses is included in **Supplementary Table 1**.

Overall, these results suggest that the basis of an individual immune response is both heterogeneous and influenced by HLA background; some subjects show large responses to just a few antigens from SARS-CoV-2 while others show a broader response. This analysis also identifies a short-list of highly immunogenic antigens to focus on for further characterization of the CD8 T-cell response across individuals.

### 3.2 Identifying shared SARS-CoV-2-associated TCRs across the population

While the diversity of TCR recombination means that most TCR responses are “private” and will be infrequently seen in other individuals, a part of the T-cell response to a disease is “public” with the same amino acid sequences observed in many individuals, particularly in shared HLA backgrounds (32). Such disease-associated TCRs can be identified using a case/control design, as previously described for cytomegalovirus (27).

To this end, a dataset of 1,015 samples from individuals currently or previously infected with SARS-CoV-2 were collected as part of the ImmuneCODE project (19). Immunosequencing was performed to sample the TCR repertoires as described in the Materials and Methods. Additionally, 3,500 repertoires from our database processed prior to March 2020 were identified as controls (see **Supplementary Table 5** for cohort summaries; for this preliminary analysis, we used the data available in the version 002 ImmuneCODE release). A lower T-cell fraction (suggesting lymphopenia) was observed in a number of the COVID-19 cases compared to healthy immune repertoires consistent with prior reports (33) (**Supplementary Figure 1**). Public COVID-19 associated TCRs, which we call “enhanced sequences”, were then identified using Fisher’s exact test, as described in the Materials and Methods.

**Figure 3:**
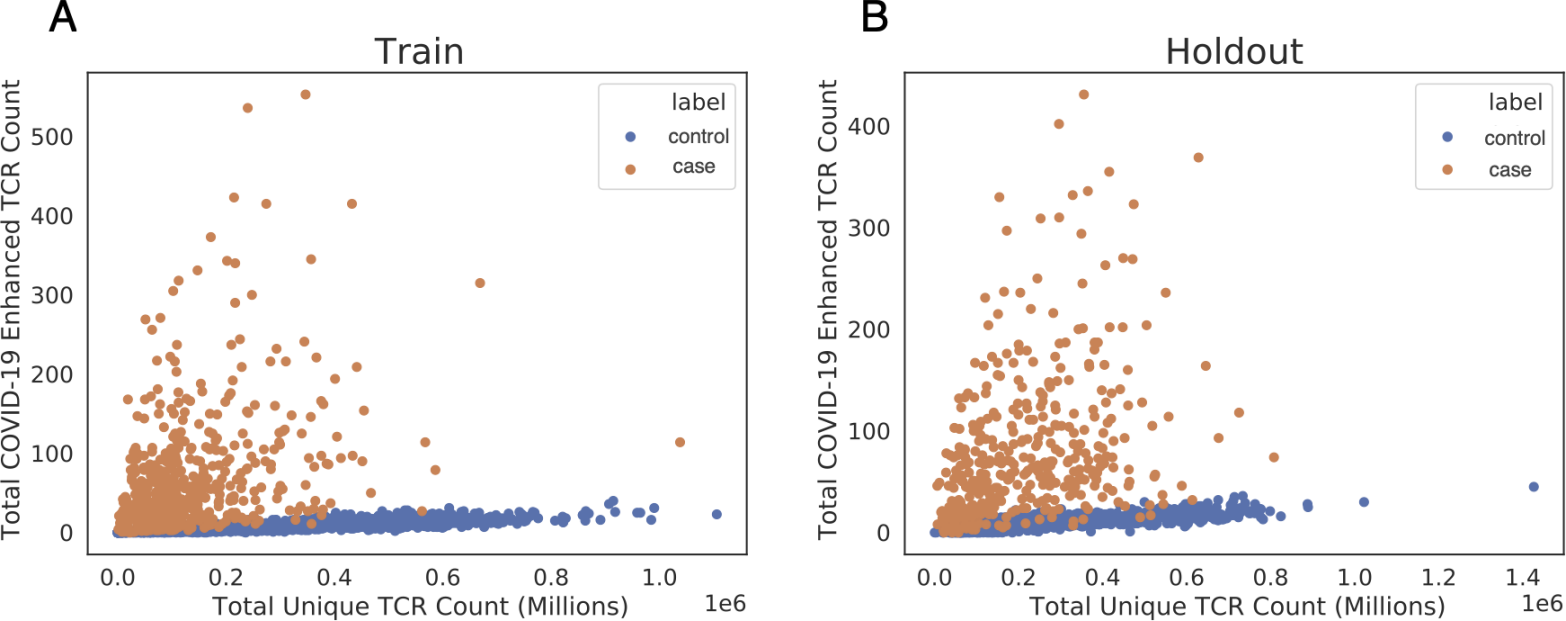
Public enhanced sequences associated with SARS-CoV-2 infection distinguish cases from controls. Each panel shows the number of TCRβ DNA sequences sampled from each subject for a large number of cases and controls. **(A)** Samples from the training set used to identify this list of enhanced sequences (DLS and NIH/NIAID cohorts). **(B)** Samples from a hold-out set with no overlap with the training set (ISB, H12O and BWNW cohorts). Both panels show a similar number and separation of enhanced sequences in cases versus controls.

As a pilot study, enhanced sequences were identified using two cohorts, DLS (from New York, USA) and NIH/NIAID (from Italy), comprising a total of 483 cases, with 1,798 pre-March 2020 controls. A total of 1,828 enhanced sequences were identified from this first dataset which collectively distinguish cases from controls (**Figure 3A**). To establish high confidence in the enhanced TCR sequences identified for SARS-CoV-2, sequence identification was also performed independently for each of these two cohorts. A total of 309 enhanced sequences from the earlier set of 1,828 were identified independently across both studies. This degree of overlap in distinct populations demonstrates the generality of the signal that has been discovered, while also pointing to the opportunity that additional data have to identify more SARS-CoV-2 associated sequences. Notably, these enhanced sequences were also substantially enriched in our other held-out cohorts in this initial dataset, which totaled 397 cases from three additional cohorts (ISB, H12O and BWNW) and 1,702 additional controls (**Figure 3B**).

If these public associated enhanced sequences are SARS-CoV-2 specific, then a subset of them should overlap with the antigen-specific TCRs identified by the MIRA experiments. We identified a total of 368 exact matches to 59 different enhanced sequences from the set of 1,828 identified above. There were also 810 matches (from 394 distinct TCRs) to sequences that were only one amino acid change away (with identical V-gene and J-gene assignments) from 68 distinct enhanced sequences. Of the 59 different enhanced sequences with any exact matches, 36 (61%) were mapped to the HTTDPSFLGRY peptide from ORF1ab, with the remaining 23 mapping to 11 other antigen locations from across the proteome including two other ORF1ab addresses, four surface glycoprotein addresses, two nucleocapsid phosphoprotein addresses, and one each from ORF6, ORF10, and the envelope protein (see **Supplementary Table 1**). Including near neighbors and other sequence-based clusters of TCRs would expand this count.

### 3.3 Public disease-associated TCRs predict the breadth and depth of the antigen-specific T-cell response

To further explore the relationship between public disease-associated TCRs and largely private antigen-specific TCR datasets identified by MIRA, repertoire sequencing was performed on the COVID-19 subjects with MIRA data using the Adaptive immunosequencing assay. Although the current MIRA experiments are limited to CD8 T cells specific to the 545 HLA-I presented peptides in the MIRA panel, intersecting a subject’s MIRA-mapped TCRs with their immunosequenced repertoire provides a lower bound estimate on the proportion of T cells in a subject that have likely expanded in response to SARS-CoV-2. Two specific quantities are of interest: the clonal breadth of the TCR repertoire, defined as the proportion of all unique TCR (DNA) clones that are SARS-CoV-2 specific (Equation 1); and the clonal depth of the TCR repertoire, related to the overall proportion of T cells that are SARS-CoV-2 specific (Equation 2).

**Figure 4:**
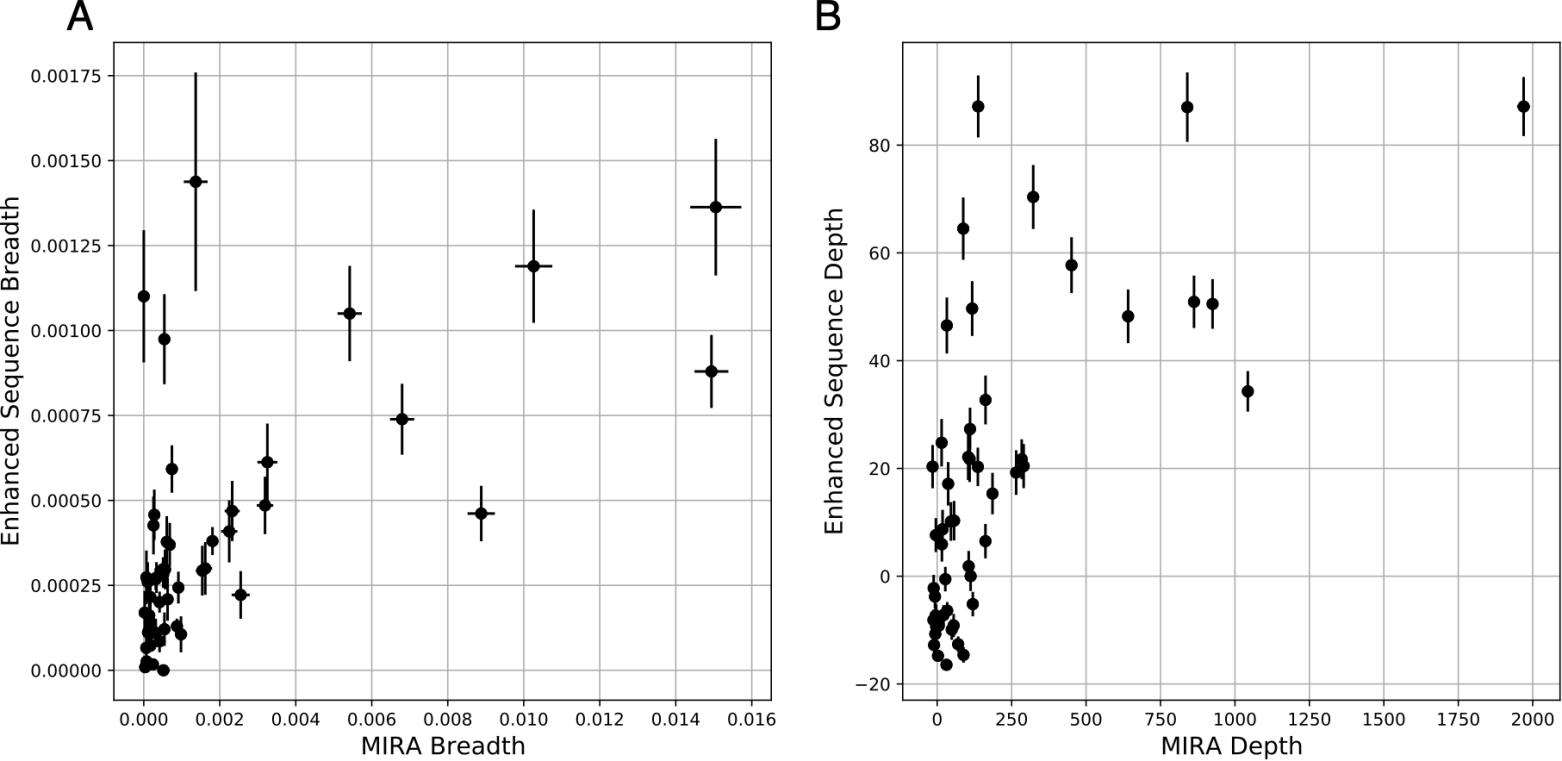
Clonal breadth and depth of the SARS-CoV-2 specific T-cell response can be estimated from MIRA-based profiling and from public enhanced sequences. **(A)** Breadth (relative fraction of unique TCRβ DNA sequences) of TCRs assigned as MIRA or enhanced sequence TCRs. **(B)** Depth of the same TCRβ DNA sequences, defined as the summed logarithm of productive template counts across all SARS-CoV-2 associated clones from the two approaches, normalized by subtracting the logarithm of total template counts across all clones. In both panels, error bars on x and y represent the standard deviation.

Across 51 samples with paired immunosequencing and COVID-19 MIRA data, we observed a remarkable concordance between either the breadth (**Figure 4A**; Spearman rho = 0.62, p = 2e-6) or depth (**Figure 4B**; Spearman rho = 0.67, p = 6e-8) of an individual’s antigen-specific response as estimated by MIRA and that of the disease-specific response as estimated through public enhanced sequences. Notably, both clonal depth and breadth as measured by an individual’s MIRA response is typically an order of magnitude higher than that estimated by public clones, highlighting the extent to which MIRA is able to identify disease-associated TCRs, in addition to mapping TCRs to specific antigens. Nevertheless, for a small number of subjects, the clonal breadth and depth as estimated by public disease-specific clones is substantially higher than what is estimated by MIRA, likely indicating the role of CD4 T cells as well as CD8 T cells specific to antigens not included in the panel.

MIRA-identified TCRs from an individual experiment are largely private (**Supplementary Figure 2**), but the scale of data from MIRA should enable identification of antigen-specific TCR patterns that generalize to new individuals (9, 10). While those efforts advance, the high concordance between public enhanced sequences and MIRA defined breadth and depth provides a useful means of estimating these quantities in large populations.

### 3.4 Analyzing T-cell response dynamics to SARS-CoV-2

**Figure 5:**
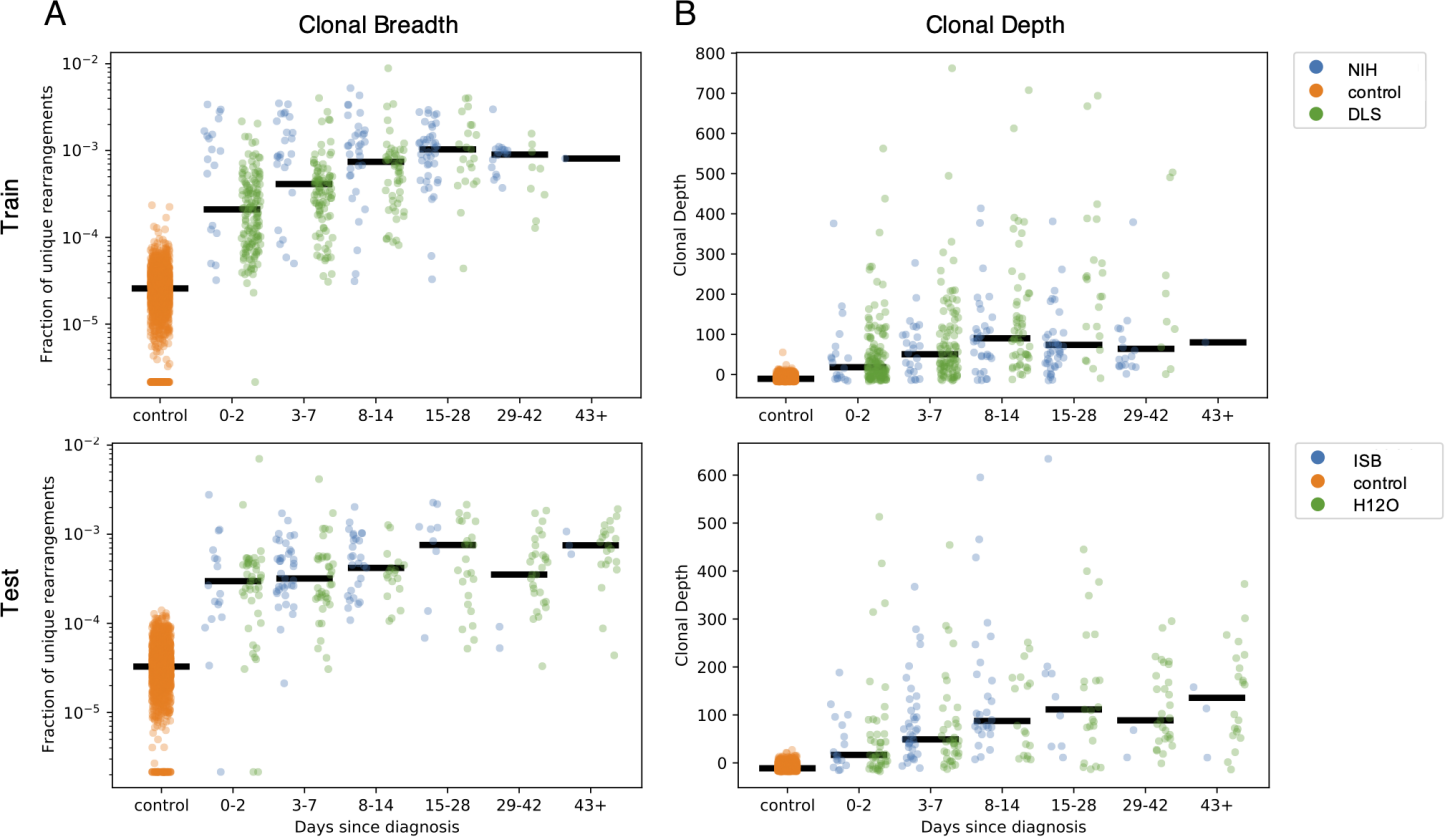
Breadth and depth of the immune response during SARS-CoV-2 infection and after recovery. The panels represent, by time from diagnosis by a viral RT-PCR test, **(A)** the clonal breadth (relative number of enhanced sequences observed) and **(B)** the clonal depth (a measure of frequency based on the summed logarithm of productive template counts normalized by subtracting the logarithm of total template counts).

As the T-cell response typically expands in the days following infection, then contracts to a steady memory state following clearance of viral antigens, the clonal breadth and depth should follow a similar trajectory. To test this hypothesis, the 1,015 COVID-19 subject samples were binned based on days since PCR-confirmed diagnosis with separate plots shown for the training and holdout sets used to discover this set of enhanced sequences (**Figure 5**). As expected, both breadth and depth indicate significant expansion of the T-cell response in the majority of subjects at time of diagnosis relative to healthy controls. As time progresses, both breadth and depth increase, reaching a peak in the 8–14 day and 15–28 day bins, then contracting slightly. Notably, both the 29–42 day and 43+ day bins show noticeably higher SARS-CoV-2-specific breadth and depth compared to controls, indicating the public enhanced sequences persist following presumed antigen clearance.

### 3.5 Public enhanced TCR sequences are highly specific in diagnosing current and past SARS-CoV-2 infection

The significant expansion in SARS-CoV-2 specific clonal breadth and depth indicate that public enhanced sequences can detect past or present SARS-CoV-2 infection. Therefore, a simple logistic regression model was trained based on clonal breadth to separate cases from controls. As above, we initially used the DLS and NIH/NIAID cohorts, with a subset of controls, for model training and then tested on a holdout set of 325 samples from 276 distinct subjects from the ISB, H12O, and BWNW cohorts (with days from diagnosis information) and 1,702 pre-COVID-19 negative controls from other cohorts. Overall, the model was highly sensitive and specific in diagnosing current or past SARS-CoV-2 infection (**Supplementary Table 6**). Using a target specificity of 99.8% across the 1,702 controls, the classifier demonstrates 77.4% sensitivity at 0–2 days post diagnosis (dpd) and 89.6% sensitivity at 3–7 dpd, further rising to 100% at 8–14 dpd. Notably, there is some reduced signal at 2–4 weeks from diagnosis; preliminary evidence suggests the negative cases are predominantly severe COVID-19 subjects who subsequently died or were in the ICU during the course of their illness, although further characterization with additional clinical/treatment data is required. The sensitivity for this first model is around 92–94% over a month after diagnosis (29+ dpd). We also investigated the model’s performance on later convalescent samples. From a separate set of 49 subjects whose blood was drawn ranging from 0–1 months, 1–2 months, and 2+ months from end of symptoms, there was ~90% sensitivity across all three of these time ranges suggesting a persistent T-cell signature after clearance of infection. The model performance is also robust to potential confounders such as age and sex (**Supplementary Figure 3**).

Both the enhanced sequence identification and logistic regression parameter estimation should improve with additional training data. Therefore, using repertoires from an additional cohort, IRST, as well as additional data from the H12O and NIH/NIAID cohorts that are part of the version 003 ImmuneCODE release, we trained a new classifier with 1,421 unique cases and 2,447 controls. The cases include all the COVID-19 cohorts listed in **Supplementary Table 5** except for ImmuneRACE. This model used a variation of the prior training method that filters enhanced sequences present in difficult-to-classify controls in the training data, as described in the Materials and Methods. Five-fold cross validation was used to assess performance (**Table 1**). This model identified a total of 4,242 enhanced sequences, more than double what was used in the initial model reported above, and increased sensitivity across multiple time ranges, reaching 85.1% at 3–7 days from initial diagnosis, 94.8% at 8–14 days from diagnosis, and >93% in all subsequent time bins analyzed out to 43+ days as shown in **Table 1**. For the set of 49 subjects whose blood was drawn ranging from 0–1 months, 1–2 months, and 2+ months from end of symptoms, sensitivity increased to 98%.

**Table 1:** Performance of a diagnostic model trained on all available data for 1,429 cases and 2,447 controls at 99.8% specificity.

	% Sensitivity (with 95% CI)	# Subjects
Days since diagnosis:		
0-2	74.5 (69.3-79.1)	329
3-7	85.1 (79.6-89.7)	202
8-14	94.8 (90.8-98.4)	134
15-28	93.6 (87.4-98.0)	94
29-42	92.9 (86.4-98.5)	70
43+	96.3 (90.2-100)	54
Days since end of symptoms:		
0-30	88.9 (66.7-100)	9
31-60	100 (100-100)	22
61+	100 (100-100)	18

Performance reported for the subset of samples with annotated times since diagnosis or symptoms using five-fold cross-validation.

### 3.6 Direct comparison of T-cell signature with antibody serology

To further explore the utility of this classifier, a direct comparison to serology was performed in the context of a real-world study. ImmuneRACE was a prospective virtual study that enrolled individuals who were exposed to, actively infected with, or recovered from SARS-CoV-2 infection in at least 24 different geographic areas across the United States (20). After completion of an online consent and questionnaire, whole blood, serum, and a nasopharyngeal or oropharyngeal swab were collected by mobile phlebotomists.

From the first 100 subjects who reported SARS-CoV-2 infection by a viral RT-PCR test, Adaptive immunosequencing was performed from whole blood and serology assays were performed by LabCorp using two different tests: the multi-antibody test Elecsys^®^ Anti-SARS-CoV-2 (Roche) and the SARS-CoV-2 Antibody, IgG test (LabCorp). As shown in **Table 2**, 94 subjects (94%) were called positive by the T-cell classifier, while only 90 subjects (multi-serology) and 87 subjects (IgG only) were called positive by the serology assays. Treating the prior RT-PCR positive test as ground truth, these results suggest that the T-cell-based approach described here has greater positive percent agreement to RT-PCR results than antibody serology.

**Table 2:** Relative performance of T-cell classifier versus antibody serology tests for 100 RT-PCR confirmed COVID-19 subjects.

		Multi-serology		IgG only	
		Negative	Positive	Negative	Positive
T-cell classifier	Negative	5	1	5	1
	Positive	5	89	8	86
IgG only	Negative	10	3		
	Positive	0	87		

Three 2x2 contingency tables show the agreement between each pair of tests, with the T-cell classifier having the most positive calls in agreement with RT-PCR results, followed by multi-antibody serology and then the IgG-only test.

These 100 samples were collected between ~10 and 100 days from initial diagnosis, ranging from active infection through to convalescence. As there are reports of declining antibody signals over time, including reports of seroreversion (3, 4), we investigated whether the negative serology or T-cell diagnostic calls have any specific time-based trends. No significant associations with days from diagnosis (**Supplementary Figure 4**) were seen for either approach. In evaluating differences in the testing results, we also considered potential differences in disease severity. Nearly all subjects in this comparison had multiple symptoms from COVID-19, but there was one asymptomatic subject who had two positive PCR tests days apart. This subject tested negative by both antibody serology tests but was positive by the T-cell-based classifier.

We also characterized the first 23 subjects from the “exposed” cohort from ImmuneRACE, who at the time of study enrollment reported exposure to someone with SARS-CoV-2 infection but themselves were not actively symptomatic nor diagnosed with COVID-19. As a result of the virtual study design, sample collection occurred several days to weeks following enrollment, allowing for several individuals to progress to acute infection prior to sample collection. A comparison between Adaptive immunosequencing and two antibody serology tests (same as above) was performed on these 23 subjects to determine whether antibody (B cell) or T-cell signals were present in these subjects. For serology, one subject was positive by the IgG-only assay, but none by the multi-isotype assay. In comparison, two different subjects were called positive based on the T-cell classifier, but negative by both antibody serology assays tested. Medical record review of these two subjects confirmed that they both had developed COVID-19 and had a positive RT-PCR test 4 days prior to the time of sample collection, with one subject reporting symptoms 5 days prior. While limited in total number, these results suggest that the T-cell classifier may be more capable of detecting signs of SARS-CoV-2 infection earlier, and in less severe cases, than tests that detect antibody (B cell) response.

## 4 Discussion

We have described an approach that uses fine mapping of TCR sequences to hundreds of antigens in conjunction with statistical association of over a thousand public enhanced sequences to track the breadth and depth of the cellular immune response to SARS-CoV-2. This T-MAP COVID approach utilizes a small volume (1–2 milliliters) of whole blood and is compatible with most standard collection methods. It reliably and reproducibly identifies and tracks SARS-CoV-2 specific T-cell clones soon after infection and for months after recovery for most subjects.

There are many advantages of the molecular assay presented as compared to standard techniques such as ELISpot for assessing cellular immune response. The biggest advantage is that standard functional assays require live cells and the results vary depending on how the sample was handled, stored and transported. The T-cell molecular assay used here is based on DNA, which is highly stable, and probes T cells with resolution down to 1/1,000,000 cells whereas functional assays are usually only sensitive down to 1/10,000 cells. The approach assesses T cells sampled randomly from blood and, unlike functional assays, is not restricted to reagent-limited subcompartments of the cellular immune response.

Although functional T-cell assays are challenging to perform, in the hands of experts their use has led to many important findings about the cellular immune response to SARS-CoV-2. This includes early profiles of the immunoreactivity of different pools of SARS-CoV-2 antigens to CD4 and CD8 T cells and identification of potential cross-reactive T cells to SARS-CoV-2 in healthy individuals (1, 2, 29, 30). Other studies have revealed strong associations between the T-cell response and disease severity (for review, see 34–36). Evidence has also emerged in a number of independent studies demonstrating detectable T-cell responses in PCR-confirmed individuals in mild or asymptomatic cases where serology was not initially detected or in those who later serorevert (3, 4).

This manuscript recapitulates some of these findings while also adding greater scale and resolution to the emerging picture of the T-cell response. While other antigen-stimulation approaches provide an aggregated result for how a pool of antigens may respond, MIRA allows for the simultaneous characterization of hundreds to thousands of individual antigen addresses, associating tens of thousands of TCRs to specific antigens. Here we also demonstrated that through population scale sequencing of immune repertoires, public TCR sequences to SARS-CoV-2 can be identified that collectively shed light on the shared immune response at the population level. These sequences, in combination with the MIRA data, allow characterization of disease- and antigen-specific responses including the breadth and depth of the overall cellular response to a viral infection.

Assessing T-cell responses still comes with challenges that will be addressed in future work. As previously discussed, the MIRA results described here assess the CD8 T-cell response and further work is needed to characterize the CD4 T-cell response. Also, despite including several hundred peptides in our initial CD8 T-cell panels, including some of the strongest predicted binders, these likely represent a fraction of the antigens presented in different HLA contexts. HLA diversity is a key part of the adaptive immune response; we have used large, diverse study cohorts to account for this variation, but continue to collect additional data in an effort to fully characterize rare HLA alleles. All of the samples used in this study were collected prior to September 2020, so they likely represent immune responses to the initial waves of COVID-19, prior to the availability of vaccines, largely in people who had not been previously infected with SARS-CoV-2. The effects of existing T-cell memory, either against SARS-CoV-2 or related human coronaviruses, were not directly assessed in this work.

The clinical and scientific utility of our results, which were generated early in the COVID-19 pandemic, has changed with the progression of the pandemic. When this manuscript was first posted as a preprint in September 2020, there was clear value in adding a T-cell based test to the clinical diagnostic arsenal for SARS-CoV-2. Indeed, Adaptive Biotechnologies (which employs several authors of this paper) received an emergency use authorization from the U.S. Food and Drug Administration in March 2021 for their T-Detect COVID test, which was subsequently used by a number of healthcare providers. However, a test that remains positive due to T-cell memory of past infections has limited utility once most people in the population have been infected, and T-Detect COVID is no longer offered as a product.

Mapping the cellular immune response to specific SARS-CoV-2 antigens could have other translational benefits. One potential application of this approach is to identify and track the T-cell response against immunogenic, virus-specific epitopes as a correlate of protection; for example, this could be relevant for assessing protection levels (following vaccination or prior infection) against severe infection in people with weakened immune systems. In addition, our results suggest that natural immune responses to SARS-CoV-2 include responses to current targets of vaccines, such as the surface glycoprotein (spike), but also include strong or in some cases stronger responses to antigens from other viral proteins, consistent with other reports (1, 30). Through the processing and presentation of viral peptides on class I HLA molecules, CD8 T cells can access antigenic peptides that are not available for antibody targeting. Creating vaccines designed to elicit T cell responses could provide a valuable compliment to existing vaccines, provided that the HLA diversity of presented peptides and target populations is accounted for.

Since the start of the COVID-19 pandemic, the scientific community has rapidly developed and deployed many tools to characterize the immune response to SARS-CoV-2 in an effort to aid the development of diagnostics and treatments for COVID-19. As the SARS-CoV-2 virus continues to evolve, successful management will depend on using a robust knowledge of how it interacts with the immune system to minimize the risk of severe infections and protect vulnerable populations. Through the data and assays presented in this manuscript, we aim to contribute to the growing body of scientific knowledge needed to effectively combat this virus now and in the future.

## Data Availability

The original contributions presented in the study are publicly available. This data can be found here: https://clients.adaptivebiotech.com/pub/covid-2020.
