# Supplementary material for: Magnitude and dynamics of the T-cell response to SARS-CoV-2 infection at both individual and population levels
Source: Front Immunol. 2025 Jan 7;15:1488860. doi: 10.3389/fimmu.2024.1488860 (PMC11747429; doi:10.3389/fimmu.2024.1488860)
Supplement: Supplementary file 1 [file DataSheet1.pdf]

frontiers

Supplementary Material

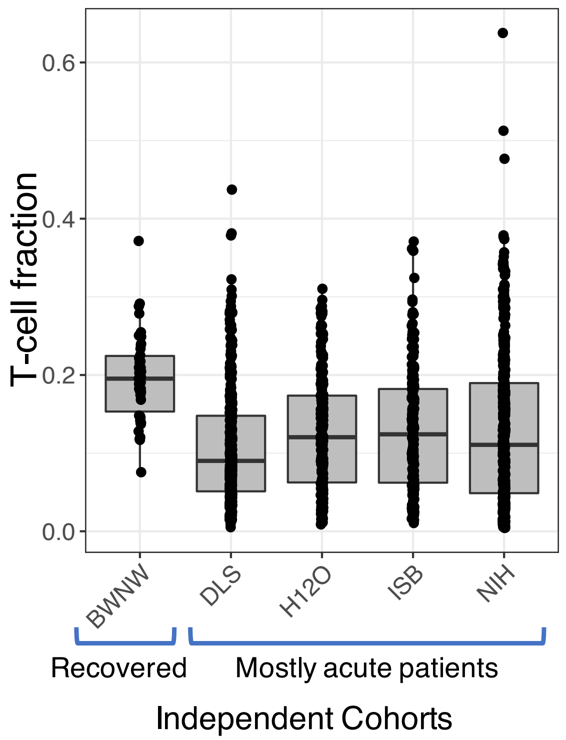

Supplementary Figure 1: Distribution of T-cell fraction across different COVID-19 cohorts.

While the samples from BWNW (exclusively convalescent subjects) are near normal values, the overall T-cell fraction is depressed across all other cohorts, which are enriched for acutely infected subjects. Individuals on the low end of these distributions would be considered severely lymphopenic.

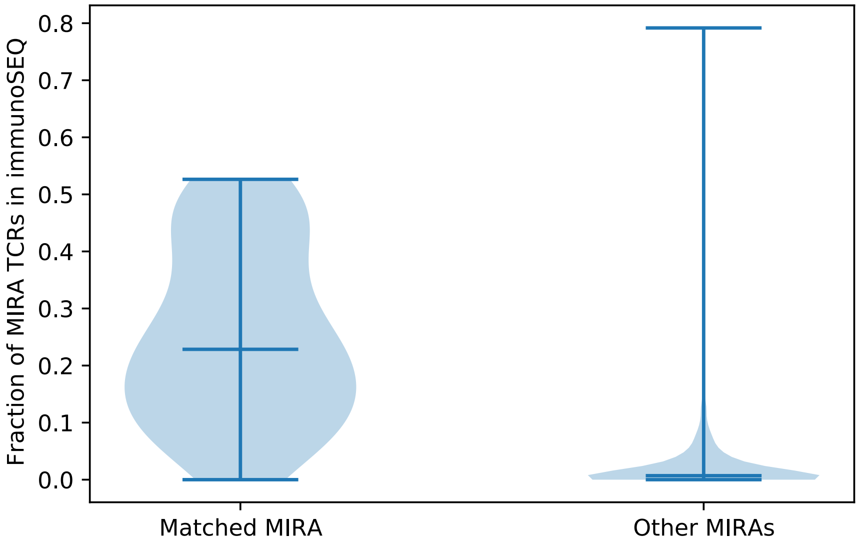

Supplementary Figure 2: Overlap of MIRA with immunoSEQ within and across subjects.

Within individuals, a median of about 25% of the TCRs identified by MIRA are detectable in a separate sample assessing the overall immune repertoire. Across individuals, this comparison drops much lower, suggesting that a majority of the detectable response is due to private TCRs.

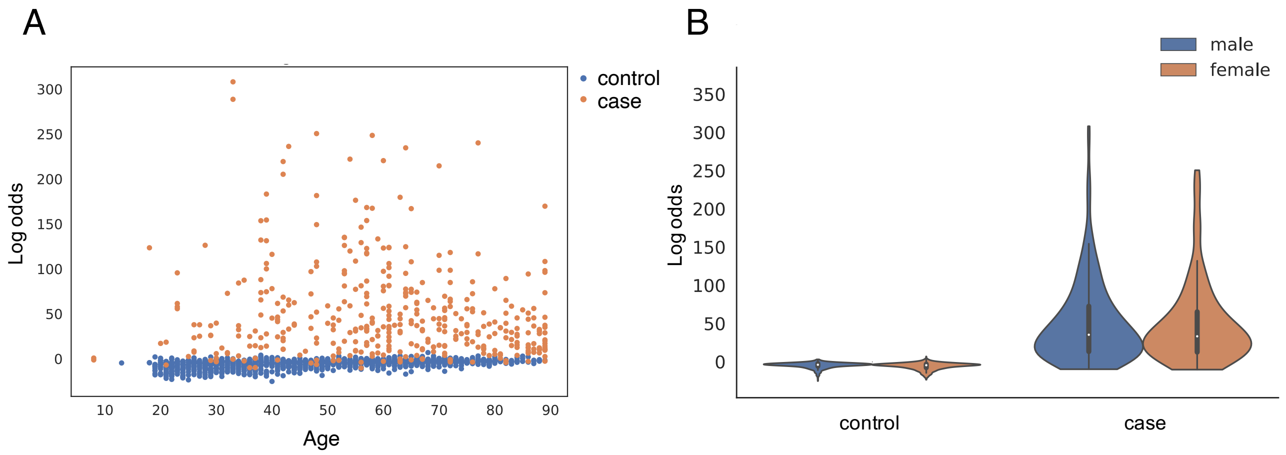

Supplementary Figure 3: Model predictions separate SARS-CoV-2 cases from controls across ages (A) and in both males and females (B).

Both plots report model scores as the untransformed log-odds estimated from the logistic regression classifier. The violin plot in panel (B) visualizes the density of log-odds scores among male and female cases and controls, with median and interquartile range values indicated.

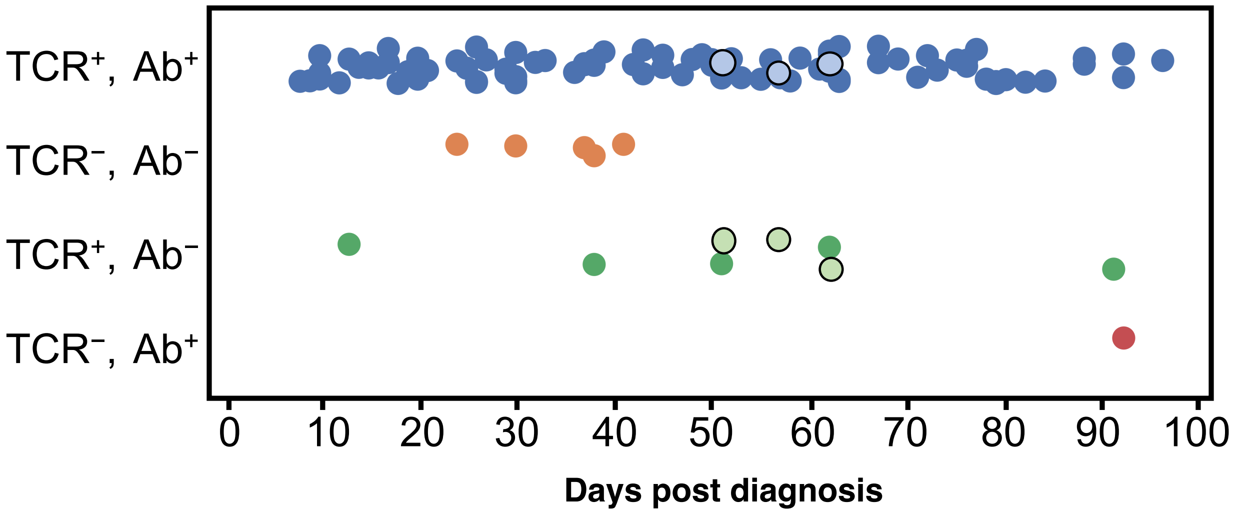

Supplementary Figure 4: Performance by time since diagnosis for the T-cell classifier and antibody serology tests for 100 RT-PCR confirmed COVID-19 subjects.

The three outlined points represent samples where the multi-antibody serology test was positive but IgG only was negative, changing the category of the points depending on which antibody test is being compared. No significant associations with time are observed for the negative calls from either the T-cell classifier or the antibody tests.

Supplementary Tables 1-4 are available as Excel files on the publisher's website.

Supplementary Table 1. Complete list of antigen locations and peptides with matches between the MIRA experiments, as well as any exact sequence matches to enhanced sequences identified in the initial case/control study.

Supplementary Table 2. Clusters of enhanced TCR sequences (by V/J gene matching and CDR3 Hamming distance within 1 AA) seen in MIRA, for eight antigens with T-cell responses in over half of MIRA experiments.

Supplementary Table 3. Enhanced TCR sequences seen in MIRA and VDJdb, for eight antigens with observed T-cell responses in over half of MIRA experiments.

Supplementary Table 4. List of antigens from MIRA data where putative HLA restrictions can be attributed based on using a Mann-Whitney U test over the number of mapped TCRs per experiment.

Supplementary Table 5. Summary of clinical cohorts included in this study, including demographic parameters

Cohort	Sample count	Subject count	Institution	Mean age (range)	% Male	Study description
**COVID-19-BWNW**	62	62	Bloodworks Northwest	54 (20, 79)	52	Whole blood samples from convalescent subjects collected at Bloodworks Northwest (Seattle, WA)
**COVID-19-DLS**	431	337	Discovery Life Sciences	70 (23, 89)	49	Whole blood samples collected during routine patient care in acute and convalescent phases procured through Discovery Life Sciences (Huntsville, AL)
**COVID-19-ISB**	157	114	Institute for Systems Biology	61 (18, 89)	42	Whole blood samples collected under the INCOVE project at Providence St. Joseph Health (Seattle, WA). Subjects were enrolled during the active phase and monitored through disease
**COVID-19-NIH/NIAID**	389	285	National Institute for Allergy and Infectious Diseases (NIAID)	45 (29, 89)	58	Whole blood samples collected in Brescia and Monza (Italy) during active infection, and provided to the NIAID (Bethesda, MD) for DNA extraction

**COVID-19-H12O**	612	570	Hospital Universitario 12 de Octubre	58 (8, 89)	29	Whole blood samples collected at the Hospital Universitario 12 de Octubre (Madrid, Spain) during the active or convalescent phase
**COVID-19-IRST**	64	53	Istituto Scientifico Romagnolo per lo Studio e la Cura dei Tumori (IRST) / AUSL-Romagna	78 (20, 89)	49	Whole blood samples collected by IRST/AUSL (Romagna, Italy) during active infection
**COVID-19-ImmuneRACE**	123	123	Adaptive Biotechnologies	43 (18, 74)	27	Whole blood samples collected from subjects from 24 geographic areas in the US with active infection, in convalescent phase, or exposed to SARS-CoV-2

Supplementary Table 6. Performance of a diagnostic model trained on an initial dataset from two independent sources and tested on a hold-out dataset of 276 distinct case samples and 1,702 pre-COVID-19 controls. Performance is reported at a level of 99.8% specificity for the classifier.

	**Holdout**		**Train (5X cross-validation)**	
	**% Sensitivity (95% CI)**	**# Subjects**	**% Sensitivity (95% CI)**	**# Subjects**
**Days since diagnosis:**				
0-2	77.4 (65.7-87.8)	63	57.3 (49.7-65.5)	164
3-7	89.6 (81.2-95.4)	79	78.3 (71.3-86.1)	125
8-14	100 (100-100)	46	88.4 (81.2-94.5)	88
15-28	81.8 (68.4-95.2)	33	90.5 (83.8-98.3)	64
29-42	93.5 (83.3-100)	31	100 (100-100)	26
43+	91.7 (78.6-100)	24	100 (NA-NA)	1
**Days since end of symptoms:**				
0-30	88.9 (62.5-100)	9		
31-60	90.9 (76.9-100)	22		
61+	100 (80.9-100)	18		

Supplementary Methods

Statistical model for TCR-antigen matching in MIRA data

The model, referred to as MIRAGE (“MIRA GEnerative model), aims to compute the posterior probability that a T-cell receptor (TCR) recognizes a specific antigen based on read counts across MIRA peptide pools.

Model assumptions:

1. **Data distribution and likelihood:** The read count data for a TCR in a pool, denoted as y, is modeled as a mixture distribution.

   When $y \neq 0$, the data is assumed to follow a log-normal distribution

   $$\log(y) \sim N(\mu, \sigma^2),$$

   where σ^2 is empirically estimated based on the mean μ using a mean-variance relationship, which is further explained in the next section.

   The probability of $y = 0$ (drop-out) is modeled as a logistic function of the mean μ,

   $$p(y = 0) = \frac{1}{1 + e^{-(\beta_0 + \beta_1 \mu)}}.$$

   Together, these equations fully describe the distribution of y conditional on μ, forming the basis for the likelihood calculation.

2. **Estimation of $\boldsymbol{\mu}$:** If a TCR binds to an antigen, we expect a particular T cell activation pattern. This activation is encoded with a pool indicator vector based on the antigen layout (*i.e.*, an indicator for presence in specific pools in which an antigen was included).

   If activated in a pool according to the antigen-specific indicator vector:

   $$\mu = \log(1e7) + \log(f),$$

   where 1e7 is the expected number of input T cells per pool, and f represents the TCR repertoire frequency, estimated using maximum likelihood.

   If not activated in that pool:

   $$\mu = \log(1e7) + \log(f) + \log(\varepsilon),$$

   where ϵ denotes a small error probability from flow sorting leakage.

3. **Mean-variance relationship:** The variance of the read counts increases with the mean. We estimate this relationship independently for each experiment. A quadratic-linear spline is used to fit the empirical data variance, approximated on the log scale with a Taylor series adjustment.

$$\sigma^2 = \frac{\mathrm{Var[y]}}{\mathrm{E[y]}^2},$$

where Var[y] is modeled as approximately linear in μ and $\mathrm{E[y]}^2$ is back-calculated from μ**.** This estimation leads to a close approximation to the empirical variance $Var[\log(y)]$ which σ^2 approximates, see the blue curve in the figure below.

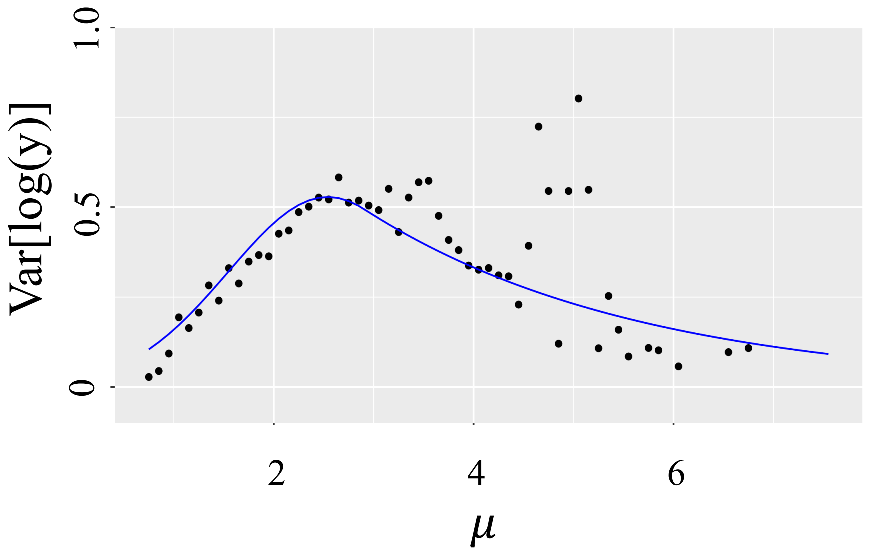

4. **Drop-out probability:** Drop-out, the event of zero reads for a TCR in a given pool, is modeled using a logistic regression approach, fitted to data from peptide-free control pools where TCRs are not expected to respond to specific antigens targeted by a MIRA experiment. This provides a probability curve for drop-out given a TCR's average read count in the sorted wells.

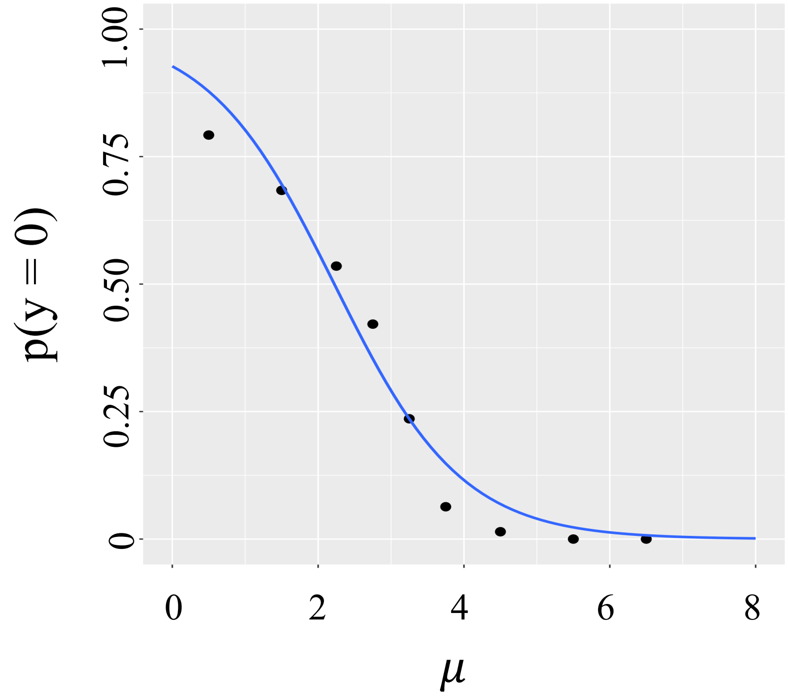

5. **Prior probabilities**: Each antigen j has a prior probability $p(z_i = j)$ that any randomly drawn TCR i binds to it. Additionally, we allow the possibility of a TCR binding non-specifically $p(z_i = NONSPECIFIC)$. These priors form a probability vector with many antigen-specific entries and one non-specific entry, constrained to sum to one. We estimate these prior probabilities using an EM algorithm to find the maximum likelihood values, making the priors data-driven in a way that aligns with Empirical Bayes methodology.

Posterior computation:

To infer the likelihood that a specific TCR binds to a particular antigen, we compute the posterior probability $p(z_i = j \mid data)$, where z_i represents the antigen that binds to TCR i. For each TCR i, we consider the possibility that it could bind to each antigen in the MIRA panel (including binding non-specifically), each associated with a specific activation pattern across pools. This means that we need to sum over all possible antigens j to account for their unique addresses in our probability estimation.

More formally, the posterior probability for TCR i binding to antigen j is given by:

$$p(z_i = j \mid data) = \frac{p(data \mid z_i = j) * p(z_i = j)}{\sum_k p(data \mid z_i = k) * p(z_i = k)}$$

Here, $p(data\ |z_i\ =\ j\ )$ denotes the likelihood of observing the read count data if TCR i binds to antigen j (calculated using the equations above in Section 1), while $p(z_i\ =\ j)$ is the prior probability of TCR i binding to antigen j. The denominator sums over all antigens k enabling us to incorporate each antigen-specific address into the posterior calculation. This method ensures that each antigen's distinct activation pattern across pools is considered, improving the accuracy of our inference on which antigen each TCR is most likely to bind to.
